# Serum estradiol/progesterone ratio on day of embryo transfer may predict reproductive outcome following controlled ovarian hyperstimulation and in vitro fertilization

**DOI:** 10.1186/1743-1050-4-1

**Published:** 2007-03-19

**Authors:** Irmhild Gruber, Alexander Just, Monika Birner, Alexander Lösch

**Affiliations:** 1 Department of Gynecology and Obstetrics, IVF Outpatient Department, General Hospital of St. Poelten, Propst Führer-Strasse 4, A-3100 St. Poelten, Austria

## Abstract

**Background:**

To determine whether estradiol-to-progesterone (E_2_/P) ratios at the time of embryo transfer (ET) have an effect on implantation and pregnancy in IVF cycles.

**Methods:**

239 women consecutively treated by IVF or ICSI were retrospectively analyzed and early luteal serum E_2 _and P were measured on the day of ET. Transfer occurred after a variable in vitro culture period ranging from 4–7 days after ovulation induction (OI). Following ET, serum E_2_/P ratios were calculated for clinical pregnancies, preclinical abortions and non-coneption cycles.

**Results:**

Receiver-operator curve analysis demonstrated that the E_2_/P ratio could differentiate between clinical pregnancies and non-pregnant cycles (area under the curve on OI +4 days = 0.70; 95% CI = 0.60–0.80; *p *= 0.003, on OI +5 days = 0.76; 95% CI = 0.64–0.88; *p *= 0.001, OI +7 days = 0.85; 95% CI = 0.75–0.96; *p *< 0.0001).

**Conclusion:**

These retrospective data may hold prognostic value regarding endometrial receptivity as reflected by E_2_/P measurements and may help improve IVF treatment outcome. Further prospective studies should be undertaken to confirm these obersveration.

## Background

Progesterone (P) and estradiol (E_2_) are required for successful conception, both to prepare the endometrium for blastocyst implantation and pregnancy. During IVF-ET, controlled ovarian hyperstimulation results in excessive follicular development and supraphysiologic serum concentrations of E_2 _and P. Such derangements raised concerns about the impact of such abnormalities on the luteal phase and a possible adverse impact on endometrial tissue [1-3]. E_2 _initiates hypertrophy and hyperplasia of endometrial epithelia, but its role in the luteal phase remains poorly understood. How E_2 _influences endometrial synchronization and blastocyst implantation is also not well described [4-6]. In contrast, the role of P in the luteal phase is better examined Csapo *et al *[7,8] showed that luteectomy leads to miscarriage in almost every case if performed before seven weeks of gestational age. P transforms the E_2_-prepared endometrium into a secretory tissue and creates a hospitable environment for embryo attachment [9].

Although previous research has established that E_2 _and P regulate events leading to implantation, relatively little is known about their relative proportion in maternal serum during the early luteal phase. In the present study, we retrospectively compared the E_2_/P ratio in the luteal phase in women undergoing superovulation for IVF-ET who had a successful implantation with those who failed to conceive after such treatment.

## Patients and methods

Records from 239 infertile patients attending the assisted reproductive unit at the Department of Obstetrics and Gynecology, General Hospital St. Poelten, Austria, from January 2003 to May 2004 were reviewed. Only those who completed the IVF/ICSI – ET cycle and had a pregnancy test in our laboratory 18 days after ovulation induction were included. Mean (± SD) patient age in this study population was 32.7 ± 3.97 years (range 18–41). Fourty-nine clinical pregnancies were achieved in 239 cycles (118 conventional IVF and 121 ICSI) after ET and a clinical pregnancy rate of 21.0 % per ET was determined. When stratified by diagnostic category, 106 patients had tubal disease (44.4%), 38 had endometriosis (5.4%), 13 had polycystic ovary syndrome (15.9%), 18 had unexplained infertility (7.5%), and 64 had male factor infertility (26.8%). For some patients, more than one infertility factor was assigned. ICSI was indicated for prior failed IVF fertilization(s) and male factor infertility as defined by severe semen abnormalities where semen analysis showed a sperm count of <20 M/mL. Patients were selected on the basis of a stimulation protocol from a computer-generated random number table.

In this study, two controlled ovarian hyperstimulation protocols were used: 61 women were treated with a conventional long protocol using a combination of intranasal buserelin (Suprecur^®^, Hoechst, Frankfurt, Germany) at a dose of 0.15 mg, 3 times daily from the midluteal phase of the cylce preceding the treatment cycle followed by rFSH (Puregon^®^, N.V. Organon, Oss, The Netherlands). Additionally, 178 women were treated with rFSH (Puregon^®^, N.V. Organon, Oss, The Netherlands) starting on day 2 of the menstrual cycle. From day 6–7 of the index cycle, 0.25 mg of ganirelix (Orgalutran^®^, N.V. Organon, Oss, The Netherlands) was administered daily as a subcutaneous injection up to and including the last day of rFSH administration. Serum concentration of E_2 _(pg/mL) and transvaginal ultrasound were used to monitor follicular growth. Ovulation was triggered by i.m. administration of 10,000 IU of hCG (Profasi^®^, Serono, Switzerland) when the mean follicular cohort diameter reached 19 mm. Ovulation induction (OI) was the beginning of the luteal phase and was designated as OI day 0. Oocyte retrieval was carried out transvaginally under ultrasound guidance 34–36 h after OI. Previous studies have described ICSI and IVF procedures in detail [1,10]. Fertilization rate was defined as the proportion of oocytes resulting in two pronuclei (2*pn*) formation; only metaphase II oocytes were counted in IVF/ICSI cycles. Transfer was carried out 4 days after OI (OI +4 days), 5 days after OI (OI +5 days) or 7 days after OI (OI +7 days). Normally cleaved embryos were replaced under ultrasound guidance using a K-soft 5001 catheter (Cook, Queensland, Australia). All patients had luteal support with Utrogestan vaginal capsules 2 × 100-mg capsules, twice a day (Viatris Pharma, Vienna, Austria) beginning on the day of embryo transfer. Patients with less than an E_2 _level <1500 pg/mL on the day of oocyte retrieval recieved only one additional luteal support of 1500 IU hCG (Pregnyl^®^, N.V. Organon, Oss, The Netherlands). Venous blood samples were collected on the morning of oocyte retrieval and on the day of ET. Serum E_2 _and P concentrations were measured via electrochemiluminescence immunoassay "ECLIA" (Roche Elecsys, Roche Diagnostics, Mannheim, Germany). For E_2_, inter- and intra-assay coefficients of variation on high concentration control (high E_2_: 1018 pg/mL) were 2.8 and 1.9%, respectively. For P (high P: 30.2 ng/mL), the inter- and intra-assay coefficients of variation were 5.5 and 2.7%, respectively. The ratio of E_2_/P was calculated for conception and non-conception cycles as defined below.

### Outcome measures

Single serum β-hCG measurement was performed on specimens obtained by peripheral veinpuncture 18 days after OI. Transvaginal ultrasound examination was performed at 8 weeks' gestation to identify clinical pregnancy, defined as the presence of a cardiac action on ultrasound scan. A conception established only on biochemical serum data was defined as preclinical abortion [11]. Supplementary P was continued until 8 weeks of gestation.

### Statistical analysis

Statistic Package for Social Sciences (SPSS v 10.0 for Windows, Chicago, IL) software was used for data analysis. Statistical significance was assessed using the Student *t*-test and χ^2 ^test as appropriate. One-way analysis of variance (ANOVA) was used to test significant difference between groups. Hormonal data were log-transformed to correct for skewness prior to statistical analysis and values in the three groups were compared using the nonparametric Kruskal-Wallis test. Significance was interpreted as *p *< 0.05. All data were presented as mean ± SD.

From this, receiver operating characteristic (ROC) curves were developed to depict probability of true-positive results (sensitivity) as a function of false-positive results (1 -specificity). Sensitivity and specificity were calculated for all determined ratios of the decision axis and combined with the area under the curve (AUC). The AUC (sensitivity/1 - specificity) format approach was used to confirm test adequacy (AUC near 1) or inadequacy (AUC near 0.5).

## Results

The means (±SD) of various clinical parameters for clinical pregnancies, preclinical abortions and for non-conception cycles are presented in Table 1. Mean basal FSH (measured on cycle day 2–4) was 7.39 ± 2.6 IU/L (range 3.0–14). Mean duration of gonadotrophin (rFSH) administration was 10 ± 1.2 days (range 7–13) for the long protocol and 10 ± 1.4 days (range 7–14) for the GnRH-antagonist protocol. The mean peak E_2 _was 1174.7 ± 828.0 pg/mL (range 164–7196), and mean number of retrieved oocytes was 8.87 ± 6.09 (range 1–28). Only one case of severe OHSS was encountered. The mean number of pre-embryos replaced was 2.5 ± 0.8 (range 1–4). The clinical pregnancy rate per ET was 18.0% (OI +4 days), 21.5% (OI +5 days) and 43.3% (OI +7 days). Fourty-nine (21.0%) had a viable intra-uterine pregnancy at 8 weeks gestations, 27 (11.2%) had an abnormal pregnancy (preclinical abortion) and 163 (67.8%) failed to conceive. There was no influence of the method of fertilization (IVF or ICSI) on the outcome (clinical pregnancies *p *= 0.668, preclinical abortions *p *= 0.564 and non-conception cycles *p *= 0.583; χ^2 ^test).

**Table 1:** Comparison between different parameters for clinical pregnancies, preclinical abortions and non-conception cycles.

	Clinical pregnancies	Preclinical abortions	Not pregnant	*P *value^a^
	(n = 49)	(n = 27)	(n = 163)	
Age (y)	32.2 ± 3.6	30.8 ± 3.3	33.1 ± 4.0	<0.05
Basal FSH (IU/L)	7.0 ± 2.6	7.2 ± 3.1	7.5 ± 2.5	NS
Peak estradiol (pg/mL)	1325 ± 816	1110 ± 586	1143 ± 864	NS
Peak progesterone (ng/mL)	8.5 ± 5	10.1 ± 6.2	8.2 ± 6.0	NS
No. oocytes/patient	10.7 ± 6.4	10.7 ± 5.7	7.9 ± 5.8	<0.05
Duration of stimulation	9.8 ± 1.3	9.8 ± 1.1	10.3 ± 1.4	NS
No. oocytes/fertilized	7.3 ± 4.3	6.9 ± 4.3	4.7 ± 3.6	<.001
No. embryo transfer	2.8 ± 0.4	2.7 ± 0.4	2.4 ± 0.9	NS
rFSH dosage (ampoules)	26.6 ± 6.8	31.4 ± 8.0	30.8 ± 10.4	NS

*Note*: Values are means ± SD. NS = not significant.

^a ^For the three groups (Kruskal-Wallis test).

There were significant differences when related to the age of the patient (*p *= 0.005), the number of oocytes retrieved (*p *= 0.002), and number of fertilized oocytes (*p *< 0.0001). There were no significant differences in basal FSH, number of gonadotropin units (rFSH) consumed and peak E_2 _and P on the day of oocyte retrieval among the three groups. The use of a GnRHa long protocol or a GnRH antagonist protocol did not alter the hormonal profile dynamics, the E_2_/P ratio or clinical pregnancy rate.

Serum (luteal) hormonal parameters at different days of ET (OI +4 days, OI +5 days and OI +7 days) and derived E_2_/P ratio for clinical pregnancies, preclinical abortions, and non-pregnant cycles are summarized in Table 2. Women with clinical pregnancies had significant higher mean E_2_/P ratios on OI +4 days (*p *= 0.01), OI +5 days (*p *= 0.005) and OI +7 days (*p *= 0.0001) compared with those who had either a preclinical abortion or failed to conceive (Table 2). Interestingly, mean serum P was higher in women with preclinical abortions compared to clinical pregnancies or non-pregnant cycles, but it did not reach statistical significance.

**Table 2:** Luteal phase characteristics of E_2_, P and the E_2_/P ratio of clinical pregnancies, preclinical abortions and non-pregnant cycles.

	Clinical pregnancies	Preclinical abortions	Not pregnant	*P *value^a^
E_2 _on OI +4 days(pg/mL)	925.4 ± 634.8	792.0 ± 586.4	812.1 ± 794.5	NS
P on OI +4 days (ng/mL)	54.0 ± 24.9	68.6 ± 60.5	56.7 ± 34.4	NS
E_2_/P Ratio on OI +4 days	18.2 ± 11.5	10.3 ± 8.5	11.8 ± 10.0	0.010
				
E_2 _on OI +5 days (pg/mL)	1612.5 ± 1118.9	1210.4 ± 576.6	1257.4 ± 905.2	NS
P on OI +5 days (ng/mL)	101.4 ± 52.6	131.5 ± 66.6	110.6 ± 56.3	NS
E_2_/P Ratio on OI +5 days	18.1 ± 11.3	9.3 ± 6.3	8.7 ± 7.9	0.005
				
E_2 _on OI +7 days (pg/mL)	2416 ± 787.9	2456.5 ± 854.9	2032.0 ± 837.4	NS
P on OI +7 days (ng/mL)	170.9 ± 57.2	187.0 ± 28.2	198.5 ± 118.4	NS
E_2_/P Ratio on OI +7 days	15.4 ± 6.2	6.5 ± 7.6	5.8 ± 10.7	<0.001

*Note*: Values are means ± SD. NS = not significant. OI = ovulation induction.

^a ^= For the three groups (Kruskal-Wallis test).

**Figure 1:**
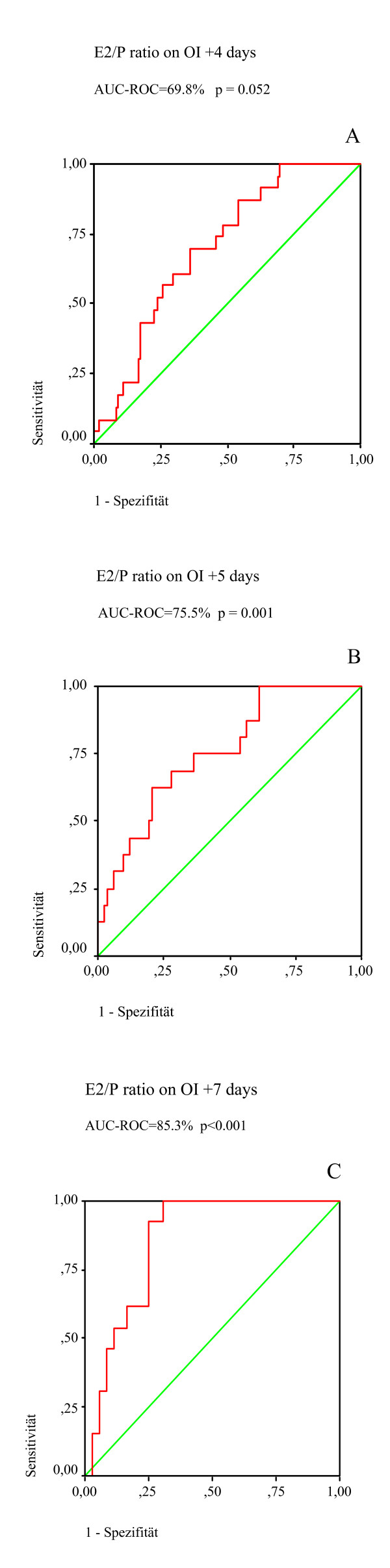
**ROC-curves for prognostic power of E_2_/P ratio**. Receiver operating characteristic (ROC) curves of the area under the curve (AUC-ROC) for prognostic power of E_2_/P ratio (**A**) on OI +4 days, (**B**) on OI +5 days and (**C**) on OI +7 days on the clinical pregnancy rate.

To analyze the prognostic power of E_2_/P ratio as measured on OI +4 days, OI +5 days and OI +7 days with respect to clinical pregnancy, the AUC_ROC _was determined with ROC analysis (Figure 1). The area under the curve suggests a relationship between E_2_/P ratio on OI +4 days (0.70; 95% CI = 0.60–0.80; *p *= 0.003), on OI +5 days (0.76; 95% CI = 0.64–0.88; *p *= 0.001) and on OI +7 days (0.85; 95% CI = 0.75–0.96; *p *< 0.0001) and the clinical pregnancy rate.

## Discussion

For normal endometrial morphology to occur, an E_2 _priming phase is required followed by P. In the pre-GnRH agonist era, the alteration of the E_2_/P ratio was considered a main cause of luteal-phase inadequacy and IVF failure, possibly mediated by the luteolytic action of E_2 _[12]. The action of estrogen is required for up-regulation of P receptors. In the follicular and early luteal phases of a normal menstrual cycle, both E_2_and P receptors are found in glandular and stromal compartements [13]. P antagonizes the proliferative effects of E_2 _on the endometrial glands by down-regulating estrogen receptors and is followed by a subsequent disappearance of P receptors [14].

Many stimulation cycles in assisted reproduction are associated with failed pregnancy despite the transfer of apparently healthy and morphologically normal embryos. This suggests impairment of endometrial differentiation or receptivity in response to E_2 _and P may also warrant consideration [15]. In our study, the role of the E_2_/P ratio at the time of embryo transfer was compared with the pregnancy outcome. Our working hypothesis of this investigation is that a high P level in combination with a low E_2 _level in the early luteal phase could presage failed implantation. These data suggest that the E_2_/P ratio on OI +4 days, OI +5 days and OI +7 days are significantly associated with clinical pregnancy rate. Interestingly, a significant higher clinical pregnancy rate could be achieved if blastocysts on OI +7 days were transferred. It seems that, blastocyst transfer allowed the identification of embryos with very high implantation potential [16], and probably a better blastocyst-endometrial epithelium interaction. The behaviour of the blastocyst may be influenced by signals from the endometrium which has been primed with preimplantation ovarian steroids [17].

Specifically, we identified no differences in peak E_2 _and P on day of oocyte retrieval or in the early luteal (on OI +4 days, OI +5 days and OI +7 days) E_2 _and P concentrations between pregnant and non-pregnant women. The use of a single luteal E_2 _and P measurement to predict endometrial receptivity was not useful, although at our center the early detection of a low E_2 _level did help identify those who were then given supplementary hCG support for corpus luteum rescue. Additionally, a single P value in the early luteal phase was not informative for diagnosing luteal phase defect. Assessment of the E_2_/P ratio in the early luteal phase provided better prognostic information with relatively higher values of this ratio being associated with a healthy corpus luteum activity and successful implantation.

In this study, patients received both GnRH-agonist and GnRH-antagonist stimulated cycles for controlled ovarian hyperstimulation to prevent premature LH surge. Uniform luteal support consisting of vaginal micronized progesterone starting on the day of ET was given to all patients in order to compensate for possible iatrogenic luteal phase defect [18]. GnRH- agonists are associated with persistent blockage of LH output for at least 10 days following the final dose [2,19]. Prolonged administration of GnRH agonists may also affect ovarian steroidgenesis directly because of the presence of GnRH receptors in the ovary [20]. In contrast, an inhibitory effect of GnRH-antagonist on steroidgenesis may also be postulated [21,22]. Here, we confirmed the present hormonal profile dynamics, and the calculated E_2_/P ratio was not affected by the treatment protocol used (GnRH agonist vs. antagonist), findings that agree with previous work [23].

## Conclusion

We conclude that moderately increased P values in the early luteal phase was associated with higher E_2_/P ratios and better pregnancy outcomes, whereas a high increase in P values in combination with a decrease in E_2_values (reflected by a low E_2_/P ratio) append to indicate poor reproductive outcome. Consequently, this retrospective study implies that in the latter setting the embryo will encounter a poorly receptive endometrium on the day of transfer, resulting in impaired implantation.

To our knowledge, this is the first study specifically evaluating E_2_/P ratio in relation to IVF outcome. Further study is needed to examine whether the E_2_/P ratios could be used as a prognostic test to predict which women will have a clinical pregnancy in the setting of advanced reproductive technologies following a COH.

## Competing interests

The author(s) declare that they have no competing interests.
